# Hepatic Gluconeogenesis and the Antidepressant Effects of Exercise: A Narrative Review

**DOI:** 10.3390/metabo16050310

**Published:** 2026-04-30

**Authors:** Hongyu Gong, Jing Miao, Jiheng Yuan, Yuchen Zhu, Huan Xiang, Yangbo Yu, Shi Zhou, Qin Zhang, Yumei Han

**Affiliations:** 1 School of Physical Education, Shanxi University, Taiyuan 030006, China; 2 College of Education and Sports Sciences, Yangtze University, Jingzhou 434200, China; 3 Faculty of Health, Southern Cross University, Lismore, NSW 2480, Australia

**Keywords:** depression, exercise, lactic acid, hepatic gluconeogenesis, liver–brain axis

## Abstract

**Background**: Research indicates that hepatic gluconeogenesis mediates metabolic coupling between the liver and muscles via the Cori cycle and participates in liver–brain axis communication through its metabolic products and regulatory networks, thereby linking it to the pathogenesis of depression. Together, these mechanisms form the molecular basis for the antidepressant effects of exercise-regulated hepatic gluconeogenesis. Regular exercise promotes skeletal muscle contraction, causing the muscles to release more lactate into the circulatory system. Lactate acts as a substrate for gluconeogenesis and activates downstream signaling pathways, thereby enhancing the gluconeogenic response. During exercise, glycogenolysis directly provides energy, while lactate produced by glycolysis enters the liver via the Cori cycle to serve as a substrate for gluconeogenesis. By maintaining blood glucose homeostasis, this process ensures a stable energy supply to the brain, thereby improving cognitive and emotional functions. This study aims to elucidate how key substrates, regulatory factors, and rate-limiting enzymes involved in hepatic gluconeogenesis and exercise influence brain energy supply, cognitive function, and emotional regulation during depression. It seeks to identify the potential targets and mechanisms through which exercise exerts its antidepressant effects via hepatic gluconeogenesis, with the goal of providing a theoretical foundation for research into the mechanisms of depression and for clinical exercise interventions. **Methods**: This review conducted a comprehensive search of the recent literature on exercise, hepatic gluconeogenesis, and depression in major domestic and international databases. Adopting an interdisciplinary approach that integrates hepatic gluconeogenesis and exercise, it synthesizes existing evidence to explore the metabolic mechanisms by which exercise improves depression through the regulation of hepatic gluconeogenesis pathways. **Results**: Research has found that exercise may modulate hepatic gluconeogenic substrates and regulate the expression of cAMP-responsive element-binding protein in states of depression, regulatory factors such as liver kinase B1, forkhead box protein 01, hepatocyte nuclear factor 4 alpha, and peroxisome proliferator activated receptor gamma co activator factor 1 alpha are used to affect key rate limiting enzymes of hepatic gluconeogenesis, such as phosphoenolpyruvate carboxykinase and glucose-6-phosphatase, enhance hepatic gluconeogenesis processes, maintain blood glucose homeostasis, ensure brain energy supply, and improve depression. **Conclusions**: Exercise intervention targeting hepatic gluconeogenesis may be a potential therapeutic strategy for depression.

## 1. Introduction

Depression is a mood or affective disorder, a mental illness caused by various reasons, with prominent and persistent low mood as the main symptom, characterized by high prevalence, mortality and recurrence rates [1], it is estimated that 5% of adults worldwide suffer from depression each year [2]. According to the World Health Organization WHO) survey report, depression has become the world’s second largest mental illness, and it is expected that in 2030, depression will rank first in the world’s burden of mental illness [3,4]. In recent years, exercise intervention has gained increasing attention as a non-pharmacological intervention for depression, offering the advantages of minimal adverse effects and low cost [5,6,7], and its mechanism may be related to the modulation of the hepatic gluconeogenesis pathway to improve energy metabolism. Exercise induced liver gluconeogenesis may be through the provision of lactate, glycerol, pyruvate and other liver gluconeogenic substrates; It regulates key metabolic enzymes of gluconeogenesis such as phosphoenolpyruvate carboxykinase and glucose-6-phosphatase in the liver; It plays an antidepressant role by improving the impaired insulin signaling and glucose transport activity, and then regulating the body’s sensitivity to insulin, which provides a new target for the intervention and treatment of depression. Based on this, this article aims to provide a narrative review of the mechanism by which exercise regulates the hepatic gluconeogenesis pathway to improve depression and compile existing evidence to provide guidance for subsequent depression research and clinical exercise intervention therapies.

## 2. Methods

This review is based on a comprehensive synthesis of the available literature and aims to explore the potential mechanisms by which exercise ameliorates depression through the regulation of the hepatic gluconeogenesis pathway. To achieve this goal, we conducted a non-systematic but comprehensive literature review. The literature searches were conducted across the following core Chinese and English databases: China National Knowledge Infrastructure (CNKI, https://www.cnki.net (accessed 20 April 2026)), VIP Database (http://www.cqvip.com (accessed 20 April 2026)), Wanfang Data Knowledge Service Platform (http://www.wanfangdata.com.cn (accessed 20 April 2026)), PubMed (https://pubmed.ncbi.nlm.nih.gov (accessed 20 April 2026)), and Web of Science (https://www.webofscience.com (accessed 20 April 2026)). Boolean operators (AND/OR) were employed to combine the following keywords: search terms in Chinese and English include “depression”, “exercise”, “lactic acid”, “gluconeogenesis”, “liver”, “energy supply” and “liver–brain axis”. Inclusion criteria were limited to original research and review articles in Chinese or English that explored these core themes, while letters, commentaries, and duplicated publications were excluded. Through the aforementioned search and reference tracing, a total of 164 Chinese and English articles were included. Given the narrative nature of this review, no formal risk of bias assessment or meta-analysis was conducted. Nevertheless, this narrative review approach enabled us to integrate findings from diverse study designs—including basic research and preclinical evidence—thereby constructing a unified conceptual framework regarding the liver–brain axis (The bidirectional, multi-path communication network between the liver and the brain) and its role in exercise-induced antidepressant mechanisms. This method is particularly well-suited for exploring emerging research fields. By synthesizing and interpreting existing evidence, it helps generate new hypotheses and research directions, thereby providing a theoretical foundation for subsequent mechanistic studies and clinical translation. This review adopts an interdisciplinary perspective that integrates gluconeogenesis and exercise, summarizing current understanding of the roles of exercise, gluconeogenesis regulators, and their key rate-limiting enzymes in the pathogenesis and intervention of depression. By synthesizing existing evidence, it further analyzes how exercise influences brain energy supply, as well as cognitive and emotional functions, through metabolic remodeling.

## 3. Hepatic Gluconeogenesis and Impaired Energy Metabolism in Depression

### 3.1. Overview of Hepatic Gluconeogenesis

**Figure 1:**
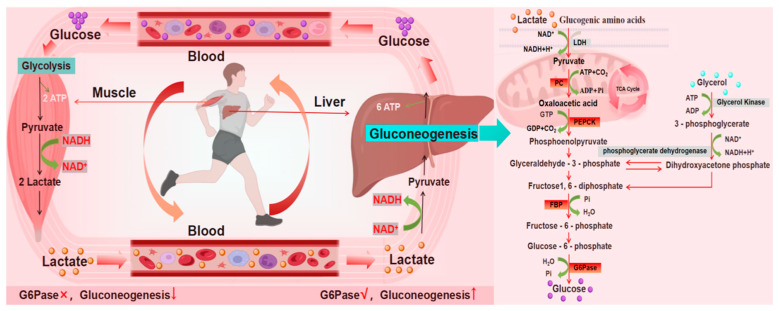
The Cori Cycle And Gluconeogenesis Process.

Hepatic gluconeogenesis is a process of glucose synthesis from non-carbohydrate precursors (lactic acid, glycerol and some amino acids). It has been found that under physiological conditions, the liver, kidney and small intestine all have some degree of gluconeogenesis [8,9,10,11]. Research indicates that the brain also exhibits a certain degree of glycolytic activity, but this is highly localized to astrocytes and may remain dormant or at low levels under physiological conditions, becoming significantly activated only under pathological conditions (such as ischemia or tumors) [12], is closely related to the pathogenesis of psychiatric disorders such as depression [13]. At the same time, hepatic gluconeogenesis is almost a reverse reaction process of glycolysis [14]. Hepatic gluconeogenesis is also involved in the Cori cycle (Figure 1) [15,16,17]. During exercise, lactic acid produced by muscle glycolysis enters the liver via the bloodstream. Under the action of lactate dehydrogenase (LDH), it is converted into pyruvate. Subsequently, through the cascade regulation of key rate-limiting enzymes such as phosphoenolpyruvate carboxykinase (PEPCK, It is the first key rate-limiting enzyme in the gluconeogenesis pathway, catalyzing the conversion of oxaloacetate to phosphoenolpyruvate and releasing carbon dioxide) and glucose-6-phosphatase (G6Pase, It is the final common key enzyme in both the gluconeogenesis and glycogenolysis pathways, catalyzing the dephosphorylation of glucose-6-phosphate to produce free glucose and inorganic phosphate), pyruvate initiates hepatic gluconeogenesis, resynthesizing glucose and releasing it into the bloodstream. This process not only sustains systemic energy demands during exercise but also G6Pase, and other key rate-limiting enzymes, initiating hepatic gluconeogenesis to resynthesize glucose and release it into the bloodstream [15,16,17,18]. This process not only maintains systemic energy homeostasis during exercise but, more importantly, the output of liver-derived glucose provides the brain with a stable energy substrate. In depressive pathologies, impaired brain energy metabolism is recognized as a significant contributor to emotional dysregulation. Thus, exercise may exert its antidepressant effects through enhanced hepatic gluconeogenesis and optimized cerebral energy supply, constituting a potential metabolic mechanism.

### 3.2. Mechanisms Underlying the Role of the Liver–Brain Axis in the Regulation of Energy Metabolism in Depression

In recent years, a large number of studies have found that the onset of depression is related to mitochondrial dysfunction and that antidepressants can play a role by interfering with mitochondrial function [19,20]. In 2011, Kato proposed the “mitochondrial dysfunction hypothesis in depression and bipolar disorder”, that is, abnormalities in mitochondrial energy metabolism play an important role in the pathogenesis of psychiatric disorders [21,22]. YangN found that improved mitochondrial function can affect energy metabolism in brain regions and effectively alleviates corticosterone-induced depressive-like behavior in mice [23]. The brain mainly relies on glucose produced energy to maintain normal physiological activities, and an imbalance in glucose homeostasis may affect the development and prognosis of a variety of metabolic diseases, which suggests that hepatic gluconeogenesis may have some relevance to energy metabolism disorders in depression [24,25]. Research has shown that lactate produced during exercise can serve as a metabolic substrate for gluconeogenesis, transporting glucose production to the central nervous system and maintaining glucose homeostasis, thereby regulating cognitive function [26,27].

**Figure 2:**
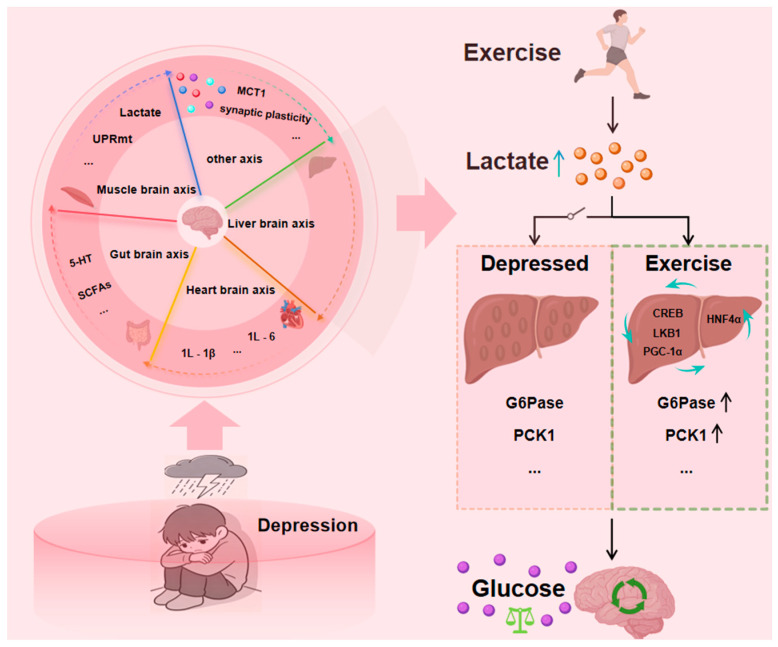
Exercise Improves Depression Through Hepatic Gluconeogenesis Interaction.

There is research available that clarified the key role of a liver–brain axis and its mediated metabolic-epigenetic regulatory pathway in exercise-induced anti-anxiety effects based on the fact that physical exercise can effectively alleviate mental disorders by improving synaptic transmission, the regulatory mechanism of anxiety disorders associated with depressive symptoms has also been revealed from the perspective of the “liver–brain axis” [28,29,30]. The metabolic and epigenetic mechanisms of the modern “liver–brain axis” are highly consistent with the Traditional Chinese Medicine theory that “the liver governs the free flow of qi and regulates emotions.” The millennia-long clinical efficacy of Traditional Chinese Medicine formulas designed to soothe the liver and dispel depression, in turn, validates that regulating liver function through the liver-brain axis can effectively influence the brain’s emotional centers. A study has confirmed that depression exists liver and brain co-morbidity pathology foundation, and liver and brain mutual influence, sparing liver may strengthen brain, wake up brain may smooth liver, and at the same time, explores “liver and brain together treats” depression mechanism [30,31,32]. Early studies also found that the heart and endocrine functions affect the functional activities of brain tissues, explaining the close connection between the heart-brain and the balance of mental activities from the perspective of the heart-brain axis [33]. From the perspective of the “liver-brain axis” the common therapeutic targets of certain liver-related diseases and brain injury pathology were investigated. The results revealed the neuroprotective effect of the liver in traumatic brain injury [34]. Chronic social frustration stress inhibits liver thioesteraseacyl CoA thioesterase 12 (ACOT12) in the liver through glucocorticoids, leading to a decrease in acetate levels and a lack of raw materials for synthesizing acetyl CoA in the brain. This results in insufficient acetylation of histones in the ventral hippocampus and decreased expression of immunosuppressive signals, leading to activation of microglia and impaired function of GABA neurons, ultimately resulting in depression like behavior. This study deepens our understanding of the mechanisms of depression and provides a potential reference for targeted acot12 intervention or acetic acid supplement intervention [35]. A preliminary study has revealed a “vagus nerve liver cortex” loop, in which the dorsal motor vagus nerve nucleusDMX promotes the production of cytokine apolipoprotein 2 in the liver through vagus efferent nerves under pressure, thereby inhibiting the activity of neurons in the medial prefrontal cortex (mPFC), leading to anxiety-like behavior [36]. The research paradigm for depression is gradually shifting from a single target to a multi-target, multi-pathway, and multi-system interaction pattern, which has given rise to cross-system theoretical hypotheses between various axes (Figure 2). These innovative theoretical frameworks provide important exploration directions for depression research. Lactic acid, as an important component involved in liver energy metabolism, has the potential to remodel energy metabolism as well as improve cognitive function with antidepressant effects. Previous studies by the group showed that lactate metabolism was abnormal in the liver, serum and hippocampus of rats in the Chronic Unpredictable Mild Stress (CUMS) model and that impaired lactate metabolism may be an important factor in the development of depression [37].

Within the framework of the liver–brain axis, the changes in liver gluconeogenic capacity can be evaluated and judged by the data of different experiments [8,37], Phosphoenolpyruvate carboxykinase (PEPCK) and glucose-6-phosphatase (G6Pase) are key rate-limiting enzymes in the hepatic gluconeogenesis pathway [9,13,38]. The expression level of key rate-limiting enzymes can usually be used as one of the auxiliary indicators to detect the ability of liver gluconeogenesis [9,39], on the cellular side, the amount of glycogen synthesized per time unit is measured to assist in detecting the ability of liver gluconeogenesis [40,41], in the animal experiment of pyruvate tolerance level or hyperinsulinemic normal glucose clamp experiment, the occurrence and development of liver gluconeogenesis function are indirectly measured [42,43,44]. It is also possible to determine the occurrence and development of hepatic gluconeogenesis by verifying changes in enzymes. In order to verify whether PCK2, one of the two isoforms of PEPCK (cytoplasmic isoform PCK1, mitochondrial isoform PCK2), has the same hepatic gluconeogenesis potential as PCK1 in the liver, Andres Mendez-Lucas combined tracer techniques with molecular biology techniques and showed that PCK2 has hepatic gluconeogenic potential in its own right and cooperates with PCK1 in regulating the Hepatic gluconeogenesis/TCA fluxes adaptations to changes in substrate or energy availability and significantly affecting hepatic gluconeogenesis and TCA cycling only in the presence of PCK1 [45]. These assessment methods provide a technical foundation for future research into abnormalities in hepatic gluconeogenesis in states of depression and how these abnormalities affect brain energy metabolism via the “liver-brain axis”.

## 4. The Role of Hepatic Gluconeogenesis in the Pathogenesis of Depression

### 4.1. The Role of Hepatic Gluconeogenesis in Energy Metabolism in Depression

Mitochondrial energy metabolism dysfunction is one of the mechanisms underlying depression [21,22]. Mitochondrial energy metabolism disorders refer to functional impairments in oxidative phosphorylation and glycolysis, as well as reduced ATP synthesis, resulting from abnormalities in mitochondrial structure and/or number. These abnormalities lead to metabolic disturbances in a series of biochemical processes, such as glycolysis and hepatic gluconeogenesis [46,47]. Pyruvate dehydrogenase plays a key role in energy metabolism, as it can convert pyruvate into acetyl CoA, which is an important metabolic substrate in the TCA cycle. Studies have found that the enzyme activity of pyruvate dehydrogenase is reduced in CUMS depressed rats, which may lead to the inability of pyruvate to enter the TCA cycle, resulting in TCA cycle obstruction and insufficient energy supply. Many studies have shown that the liver is the metabolic center of the body and is also the main site of gluconeogenesis; Mitochondria, which are rich in the liver, are responsible for providing a place for the final oxidation of sugar, fat, and amino acids [48,49]. Numerous pieces of evidence indicate that patients with depression often have metabolic pathway disorders such as glycolysis and TCA. The pyruvate lactate axis, as a metabolic hub for glycolysis and TCA, plays a crucial role in the pathogenesis of depression [36]. The liver is closely related to the pathogenesis of psychiatric disorders such as depression, and depression is often accompanied by metabolic disorders of the liver and other disorders [50,51]. Lactate not only plays an important role in glycolysis and the TCA cycle hub in the liver but also is an important substrate in the process of energy metabolism, plays an important role in the maintenance of energy metabolism homeostasis in the body, and affects a variety of physiological activities [17,36,52].

Collaborating with Professor Junsheng Tian from the research group found that most of the body’s energy comes from oxidative phosphorylation, glycolysis and hepatic gluconeogenesis in the Tricarboxylic acid cycle [52]. Stable isotope tracer metabolomics revealed that hepatic glucose metabolism was abnormal in depressed rats, as evidenced by the blockage of the TCA cycle, compensatory activation of the hepatic gluconeogenesis pathway by the body for alleviation, and the content of enzymes related to glucose metabolism, such as pyruvate kinase and pyruvate dehydrogenase, was greatly altered, which suggests that the hepatic gluconeogenesis may be activated in the depressed rats [53]. Chen Z found that, in addition to the FAM3A-deficient mice fed with HFD, activating the FAM3A signaling pathway in the liver and brown adipose tissue can inhibit gluconeogenesis and adipogenesis in the liver. In the clinical treatment of patients with diabetes combined with depression, insulin/insulin secretagogue and doxepin may simultaneously act on patients with diabetes and depression [54]. Studies have suggested that, in addition to inhibiting the hepatic gluconeogenesis pathway, there is a need to ensure that the accumulated pyruvate can be oxidized successfully so that the TCA cycle can function properly to restore normal energy metabolism [52]. The above studies suggest that impaired hepatic energy metabolism may be associated with depressive states.

### 4.2. Role of Lactate in Energy Metabolism in Depression

In recent years, studies have shown that lactate can participate in metabolism both as an energy substrate and as a signaling molecule, affecting a variety of homeostatic functions in the body [51,55,56,57]. Lactic acid is not only involved in glycolysis, but also an important substrate in energy metabolism, plays an important role in maintaining energy metabolism homeostasis in the body, and affects a variety of physiological activities. In 1947, a clinical study initially showed that oral lactic acid relieved clinical symptoms in depressed patients [58]. Subsequently in 2018, Carrard et al. reported for the first time that intraperitoneal injections of lactic acid produced antidepressant effects based on the forced swim test (FST) in mice [59]. In 2019, Karnib et al. reconfirmed an antidepressant effect after intraperitoneal injection of lactic acid based on the Chronic social defeat stress (CSDS) and social interaction test in mice [60]. Not only did peripheral lactate infusion significantly ameliorate depression, but exogenous lactate infusion to the central nervous system had the same effect. Lactate injection into SD rats undergoing lateral ventricular catheterization resulted in a significant reduction in immobility time for forced swimming, intracerebroventricular injection of lactate in SD rats significantly reduced immobility time in the forced swimming test, and this effect showed a significant correlation with both the dose and duration of lactate administration, and in this study, in this study, lactate activated cAMP signaling, upregulated BDNF expression, and promoted neuronal survival, ultimately alleviating depressive behavior in rats [61]. The above study suggests that lactate has a significant ameliorative effect on the regulation of pathological processes in depression. Meanwhile, it was found that abnormal changes in lactate levels or related metabolism were also present in the liver tissues of CUMS model rats [44,56,62].

Ling-Hu found in the CUMS model rats that the main energy metabolism characteristics of the body are the obstruction of the liver TCA cycle and the simultaneous compensatory activation of the gluconeogenesis pathway [62]. The disruption of energy substrate supply may affect the lactate metabolism pathway in depression, blocking the conversion of lactate to glucose and inhibiting its entry into the tricarboxylic acid cycle for sufficient oxidation and energy supply. Wenxia G found an increase in lactate levels in the liver of CUMS model rats, and the metabolic characteristics mainly manifested as metabolic disorders such as amino acid metabolism, carbohydrate metabolism, and lipid metabolism [63]. Our research group also found an increase in lactate levels in the liver mitochondria of CUMS model rats, with metabolic characteristics mainly manifested as mitochondrial respiratory chain disorders, reduced ATP content, and energy metabolism disorders [64]. The above research suggests that the significant increase in liver lactate levels during depression is mainly caused by disturbances in energy metabolism pathways, mitochondrial dysfunction, and lipid metabolism disorders [62,63,64]. Therefore, by regulating energy substrates such as lactate in the hepatic gluconeogenesis pathway, new therapeutic targets can be provided for the treatment of depression.

Our previous study showed that 4 weeks of short-term aerobic and resistance exercise interventions significantly modulated lactate levels in skeletal muscle and plasma and alleviated depressive behaviors in CUMS rats, suggesting that lactate metabolism may be one of the endogenous mechanisms by which exercise ameliorates depression [36,65]. Quercetin and isoquercetin have been shown to modulate hepatic gluconeogenesis pathways by affecting two key core rate-limiting enzymes of hepatic gluconeogenesis, PEPCK and G6Pase, in an in vitro experimental study of an alcoholic extract of okra, which has been shown to be informative for antidepressants [66]. There is also a study that has used Ginseng and Qi Glycoprotein Formula to regulate the long-chain non-coding RNA signaling pathway in the rat liver, by inhibiting the expression of downstream genes PEPCK and G6Pase, reducing hepatic gluconeogenesis to regulate glucose metabolism, which may indirectly ensure a sustained and stable energy supply to the brain, avoiding cognitive impairment caused by blood glucose fluctuations [67,68].

## 5. Exercise Regulates Liver Gluconeogenesis to Improve Depression

### 5.1. Regulation of Hepatic Gluconeogenesis by Exercise

**Figure 3:**
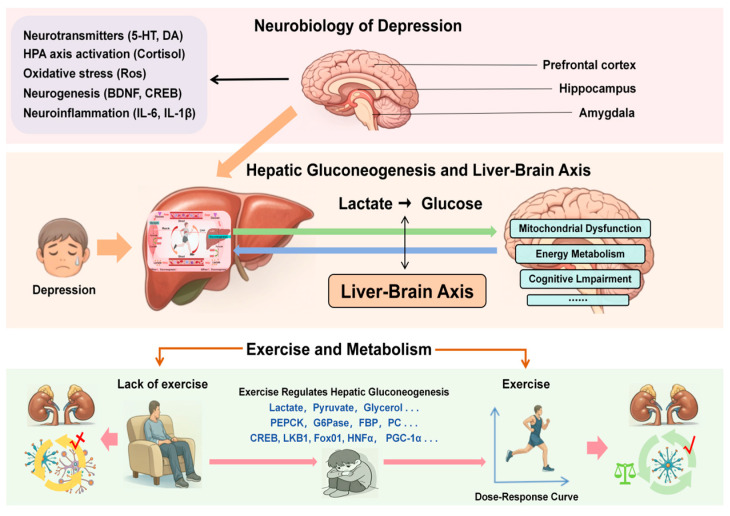
Liver-Driven Gluconeogenesis, Metabolic Dysregulation and Depression.

Exercise optimizes the production of glucose in the liver, which enhances the body’s sensitivity to insulin, indirectly supporting the maintenance of blood glucose levels within the normal range, which in turn affects the levels of neurotransmitters in the brain, such as serotonin and norepinephrine, which are closely related to the regulation of mood in the brain [69,70,71,72]. The multi-synaptic connection from MeA to the liver promotes the rapid synthesis of glucose through gluconeogenesis in the liver, thereby coordinating metabolic responses to stress. The occurrence and development of depression are often closely related to stress, and under long-term stress stimulation, it can even worsen the depressive condition. In terms of glucose supply, a more stable supply of blood glucose to the brain is needed, which is also closely related to depression [73]. The neurobiological mechanism of depression involves the dysfunction of neural circuits (prefrontal lobe, hippocampus, amygdala), neurotransmitters (serotonin, dopamine, norepinephrine) and neurochemicals (Figure 3). The pathologically related metabolites of depression include tryptophan, kynurenine, bile acids, short-chain fatty acids, etc. Liver gluconeogenesis can maintain the blood glucose supply. The efficiency with which lactate is converted into glucose via gluconeogenesis determines the ability to maintain blood glucose homeostasis during exercise, but also directly affects the stability of energy substrate supply of the brain, a high-energy-consuming organ. Moderate regular exercise can optimize liver gluconeogenesis and regulate neurotransmitters. Insufficient exercise will aggravate liver gluconeogenesis and lactic acid accumulation; Excessive exercise can overstimulate the HPA axis, leading to pathologically elevated levels of cortisol—the primary positive regulator of hepatic gluconeogenesis. This elevation may abnormally upregulate gluconeogenic flux, thereby disrupting the steady supply of hepatogenic metabolites (such as glucose and lactate) to the brain. This imbalance in the peripheral-central metabolic coupling may exacerbate oxidative stress in the brain and inhibit hippocampal neurogenesis, representing a potential mechanism by which excessive exercise affects brain energy metabolism via the liver–brain axis [18,70,74,75]. Excessive exercise also leads to a massive accumulation of lactate in muscles. Muscle cells secrete lactate bodies, which travel to the liver via the bloodstream, thereby affecting the substrates for hepatic gluconeogenesis and disrupting normal hepatic metabolic function, ultimately inducing hepatocyte apoptosis [76].

A study has shown that immediately after intense exercise, blood tends to stagnate in peripheral skeletal muscles. The compression effect of skeletal muscles during recovery can accelerate the return of blood from motor organs to internal organs, promote local blood circulation, optimize liver gluconeogenesis levels, and have a positive effect on improving depression by enhancing brain blood flow and oxygen supply, regulating stress and metabolism [77]. Exercise under fasting conditions appears to upregulate genes associated with hepatic gluconeogenesis and mitochondrial biogenesis, suggesting that longer exercise training may have enhanced hepatic mitochondrial adaptations [78]. After exercise, lactate is converted to pyruvate by lactate dehydrogenase (LDH) in the liver, which is catalyzed by pyruvic carboxylase (PC) to oxaloacetate after entering the mitochondria and then enters hepatic gluconeogenesis via PEPCK; pyruvate can also be oxidized and decarboxylated to acetyl-coenzyme A to enter the tricarboxylic acid cycle (TCA) to generate a high-energy electron carrier to supply the electron transport chain (ETC) which drives oxidative phosphorylation to produce ATP [79]. The ATP produced by the TCA cycle and the electron transport chain serves as the energy source for gluconeogenesis (a catabolic process that requires 6 molecules of ATP to produce each molecule of glucose), while substrate flux and hormonal regulation act as the regulatory mechanisms for gluconeogenesis (determining when the process occurs and at what rate). In addition, exercise also exerts effects on hepatic gluconeogenesis that optimize energy supply, substrate metabolism regulation, and long-term adaptive effects [80,81].

It is worth noting that the improvement of liver gluconeogenesis and energy metabolism through exercise is not an isolated peripheral event, but an important link in its systemic antidepressant effect. Research has shown that exercise improves insulin sensitivity, which can be transmitted to key brain regions to protect their cognitive function [82]. In some animal experiments, a series of methods including the establishment of animal models, behavioral tests, determination of biochemical indexes, and the application of molecular biology techniques are often used to observe the behavioral changes in depression model animals through regular exercise training, and combined with biochemical and gene expression analyses of liver tissue samples, to reveal the specific mechanisms of exercise on the pathways of hepatic gluconeogenesis and energy metabolism [83,84]. Research has found that exercise improves liver insulin sensitivity and regulates glucose metabolism, creating a stable physiological environment for the brain and avoiding emotional disorders such as anxiety caused by blood sugar fluctuations [85,86]. Donovan found that after exercise, skeletal muscle uptake of glucose decreased, a phenomenon confirmed in both humans and rats, which may help prevent excessive blood glucose consumption after exercise; Exercising individuals (at least in rats) significantly enhanced liver gluconeogenesis ability [87]. This adaptive change is crucial for maintaining blood glucose homeostasis. Due to the high dependence of the brain on glucose as an energy substrate, stable blood glucose levels can ensure energy supply to key emotion-regulating brain regions such as the prefrontal cortex and hippocampus and may regulate depressive like behavior by stabilizing signals such as insulin and brain-derived neurotrophic factor [88,89,90].

### 5.2. Regulation of Hepatic Gluconeogenic Substrates by Exercise

Hepatic gluconeogenesis takes place mainly in the liver [91,92], through non-carbohydrate substrates such as glycerol, lactate, pyruvate, and gluconeogenic amino acids into glucose to maintain glucose levels and energy balance in the body [93,94]. Hepatic glucose production accounts for 90% of endogenous glucose production [95]; it is essential for the maintenance of systemic glucose homeostasis [9,96]. During exercise, skeletal muscles produce a large amount of lactate due to high-intensity contractions, resulting in a significant increase in lactate concentration in the blood [36]. This change not only provides sufficient hepatic glucose precursors but lactate itself is also a key metabolic signal. Monocarboxylate Transporter 1 (MCT1), as the main channel for transmembrane transport of lactate and other precursor substances, may be upregulated in its expression and activity during exercise adaptation, thereby efficiently transporting some lactate from muscles to the liver. After the liver takes up lactate, it is converted into glucose as a hepatic gluconeogenic substrate [97,98], regulate the energy metabolism process of the body; On the other hand, the lactate metabolism process collaborates with hormone signals such as adrenaline and glucagon induced by exercise to upregulate MCT1 expression, activate PEPCK and G6Pase genes, and enhance liver gluconeogenesis [99,100], to meet the sustained energy demand of the body during exercise.

During exercise, large amounts of hepatic gluconeogenesis precursors such as lactate are produced in skeletal muscle. Monocarboxylate Transporter 1 (MCT1), a member of the MCT family, is the main channel for the transmembrane transport of precursors such as lactate, which is converted by transporting the substance from the muscle to, and extracting it from, the liver and undergoing hepatic gluconeogenesis to glucose [97,98] regulates energy metabolism in the body. By upregulating MCT1 expression, activating PEPCK and G6Pase genes, and initiating hepatic gluconeogenesis [99,100].

#### 5.2.1. The Regulatory Effect of Exercise on Lactate

Lactate is a hepatic gluconeogenesis precursor that is released from skeletal muscle during exercise and is taken up by the liver and converted to pyruvate, which is also used for hepatic gluconeogenesis [101,102]. When peripheral blood lactate levels increase and form a concentration gradient with brain lactate levels, MCT-mediated transport at the blood–brain barrier [103]. One study quantified the contribution of hepatic gluconeogenesis to glucose production during exercise, especially near the lactate threshold, using isotope tracing techniques (labeling lactate and glucose), during exercise (especially near the lactate threshold) by isotopic tracer techniques (labeled lactate and glucose), yielding the key finding that the rate of hepatic gluconeogenesis increases significantly when blood lactate concentrations increase (when exercise intensity reaches the lactate threshold), suggesting that the enhanced availability of lactate as a hepatic gluconeogenic precursor directly drove the upregulation of hepatic gluconeogenesis levels [104,105]. An early investigation of liver energy charge in mice after a single exercise session found a significant increase in adenosine monophosphate (AMP) and a strong decrease in ATP in the liver [106]. Using infusion studies with isotopic tracers of lactate, glycerol, and fatty acids, researchers have demonstrated the importance of lactate as a major gluconeogenic precursor substance while proving the mechanism of lactate shuttling from a variety of tissues and cellular aspects [107]. And hepatic glycogen is replenished during the postprandial absorption period, and this replenishment is partly due to increased hepatic uptake of hepatic glycogen and partly due to the increased glycolytic flux of glycogen [108]. In summary, exercise affects hepatic gluconeogenesis by modulating lactate metabolism, thereby facilitating the conversion of lactate to glucose as a substrate and maintaining energy homeostasis.

#### 5.2.2. The Regulatory Effect of Exercise on Other Substrates

Exercise induces the release of glycerol from skeletal muscle and adipose tissue into the circulation and alanine from muscle, both of which serve as hepatic gluconeogenesis precursors during exercise [109]. During hepatic gluconeogenesis, higher levels of glycogen were found to be produced by glycerol as a substrate in macrophages, and one possible explanation for this finding by the investigators is that the process involves fewer enzymatic reactions and that glycerol may be converted to glycogen more rapidly [110]. Exercise activates hepatic gluconeogenesis by facilitating the muscular delivery of gluconeogenic precursors (pyruvate, glycerol and gluconeogenic amino acids) to the liver and enhancing the hepatic uptake and glucose conversion of these precursors [98]. Pyruvate is an important precursor of hepatic gluconeogenesis, and an animal study injected with exogenous pyruvate demonstrated that exogenous pyruvate promotes hepatic gluconeogenesis to glucose conversion, a precursor substance that serves both as a source of blood glucose release and synthesizes glycogen for storage [111]. Exercise (especially endurance exercise) enhances gluconeogenic activity and glycerol supplementation supports energy supply as an effective gluconeogenic substrate, which is essential for maintaining blood glucose stability [112].

During high-intensity exercise, skeletal muscles produce a large amount of lactate, which provides energy substrates for hepatic gluconeogenesis through the Coricycle. During low-intensity exercise, adipose tissue breaks down to produce glycerol, which provides energy substrates for hepatic gluconeogenesis through the adipose liver muscle axis. In an animal experiment, researchers had mice run slowly and quickly on a treadmill, and compared to mice in a resting state, the glycerol concentration in the blood of mice running slowly increased; The lactate concentration in the blood of mice running quickly increased, indicating that the liver may use different matrices for hepatic gluconeogenesis during different intensities of exercise [18]. In addition to the energy substrates for hepatic gluconeogenesis, micronutrients such as selenium and iron in the blood are also key factors that directly or indirectly regulate hepatic gluconeogenesis [113,114,115,116].

### 5.3. Regulation of Hepatic Gluconeogenic Metabolizing Enzymes by Exercise

Hepatic gluconeogenesis is an important part of glucose metabolism in the liver and is regulated by several transcription factors. For example, Fox01, peroxisome proliferator-activated receptor gamma coactivator-1 alpha (PGC-1α), hepatocyte nuclear factor 4 alpha (HNF4α), Liver kinase B1 (LKB1), cyclic adenosine monophosphate response element binding protein (CREB) are key transcription genes in the hepatic gluconeogenesis signaling pathway [38,117,118]. These enzymes all play important roles in the process of hepatic gluconeogenesis [119,120,121]. Some studies have also found a close relationship between C/EBP-α and C/EBP-β and hepatic gluconeogenesis after cerebral ischemia, and they, along with Fox01, may be involved in the regulation of hepatic gluconeogenesis-associated target genes in cooperation with other transcriptional coactivators, such as CREB and PGC-1α [122,123]. Similarly, the insulin signaling pathway and the LKB1 (serine/threonine kinase)/protein kinase (AMPK) pathway are the two main signaling pathways that inhibit hepatic gluconeogenesis. The insulin signaling pathway is mediated primarily through the serine/threonine protein kinase Akt, which phosphorylates and inactivates PGC-1αand the Fox0 family in mammalian livers, mainly Fox01 and Fox03 [124,125]. The LKB1 (serine/threonine kinase)/protein kinase (AMPK) pathway acts as an important endogenous inhibitor of hepatic gluconeogenesis [126]. LKB1 is the main upstream kinase responsible for activating the AMPK loop and triggering a series of reactions to achieve energy homeostasis; and in addition to AMPK, some LKB1-dependent kinases can also indirectly inhibit hepatic gluconeogenesis through CRTC2 phosphorylation, which inhibits hepatic gluconeogenesis by phosphorylating common downstream substrates [123,127,128].

Normally, insulin inhibits hepatic gluconeogenesis by phosphorylating Fox01, and specific knockdown of the Fox01 gene in the livers of insulin-signaling-blocked mice resulted in the maintenance of normal hepatic gluconeogenesis levels in mice [129]. Exercise promotes the expression of PGC-1α and activates PEPCK and G6Pase, the key enzymes of hepatic gluconeogenesis, thus increasing hepatic gluconeogenesis. However, different factors, such as exercise duration, exercise frequency, and exercise intensity, can affect and differentially affect the specific expression ofPGC-1α, suggesting that PGC-1αis an important indirect regulator of hepatic gluconeogenesis [130,131]. Exercise also has a role in the regulation of such transcription factors [132,133,134]. In addition, Weina Liu’s team also investigated the important role of skeletal muscle PGC-1αin exercise antidepressant and its mediating mechanism and proposed the PGC-1α-mediated peripheral-central “dialog” model [135].

In a study of 8 weeks of aerobic exercise on hepatic gluconeogenesis and its signaling regulation in IR mice, the results indicated that long-term high-fat dietary intervention led to a significant increase in the content of PEPCK and G6Pase, key enzymes of hepatic gluconeogenesis, which resulted in an obvious increase in hepatic gluconeogenesis, whereas the content of the key enzymes of hepatic gluconeogenesis significantly decreased after 8 weeks of aerobic exercise intervention, which led to a decrease in hepatic gluconeogenesis and the inhibition of hepatic gluconeogenic key enzymes, such as PEPCK and G6Pase expression and improved liver function [118]. In the fasting state, glucagon secretion is increased, which decreases insulin levels, and glucagon activates cyclic adenosine monophosphate-protein kinase A (cAMP-PKA) signaling to mediate activation of the regulatory gene transcription factor CREB, which facilitates PEPCK and G6Pase transcription of PEPCK and G6Pase [136,137]. In addition, it has been found that exercise promotes neural remodeling and functional recovery in rats by modulating the PKA/CREB signaling pathway [138].

In a study on the hepatic gluconeogenesis mechanism, the cascade regulation of ZBED3 (zinc-finger BED structural domain protein 3) has been shown to affect the phosphorylation of CERB and promote the expression of downstream hepatic gluconeogenic genes to promote hepatic gluconeogenesis [139]. In addition, fructose-1,6-bisphosphatase (FBP), a rate-limiting enzyme of hepatic gluconeogenesis with two isoforms, FBP1 and FBP2, catalyzes the hydrolysis of fructose-1,6-bisphosphate to fructose-6-phosphate [140]. PC is a key enzyme that catalyzes the carboxylation of pyruvate to produce oxaloacetate and is the first rate-limiting step in hepatic gluconeogenesis [141,142].

The key rate-limiting enzyme in hepatic gluconeogenesis, PEPCK, also known as PCK [143,144], it utilizes pyruvate as the precursor substance of the process and catalyzes the generation of phosphoenolpyruvate through the oxaloacetate-aspartate or malate pathway, which is the main point of regulation of the hepatic gluconeogenesis process [145,146]. Two forms of PEPCK are present in human tissues, the cytoplasmic (cytosolic phosphoenolpyruvate carboxykinase, cPEPCK or PCK1) and mitochondrial (mitochondrial phosphoenolpyruvate carboxykinase, mPEPCK or PCK2) forms [147,148,149]. There have been many studies on the function of PCK1, which is expressed in the liver, kidney and small intestine [16], it acts as one of the key rate-limiting enzymes in the hepatic gluconeogenesis pathway and converts oxaloacetate to phosphoenolpyruvate mainly in the cytoplasm [147]. Meanwhile, mice with reduced PCK1 expression showed insulin resistance, hypoglycemia, and hepatic steatosis, suggesting an important role for PCK1 in regulating glucose homeostasis and lipid metabolism [147,150]. By analyzing the changes in human liver specimens before and after hepatic ischemia–reperfusion injury, Zhang Qi, Xu Yan and Ge Mian at Sun Yat-sen University collaborated to find that hepatic gluconeogenesis could be blocked by PCK1 inhibitor and exacerbated hepatic ischemia–reperfusion injury, whereas overexpression of PCK1 played the opposite role, suggesting that PCK1 is a potential target to alleviate hepatic ischemia–reperfusion injury [151].

G6Pase is a key rate-limiting enzyme in the hepatic gluconeogenesis pathway [147,148]. The effects of exercise on G6Pase, a rate-limiting enzyme involved in the synthesis of glycogen and the final step of hepatic gluconeogenesis and glycogenolysis, are mainly characterized by the inhibition of G6Pase activity, and its increased expression exacerbates the imbalance of glucose metabolism [146]. PGC-1α acts as a transcriptional coactivator for the expression of the hepatic gluconeogenic enzymes G6Pase and PEPCK and is also involved in PDK4 gene activation [152,153]. Thus, G6Pase mRNA levels were found to be strongly up-regulated in the liver after acute exercise. G6Pase is essential for glucose delivery, including glucose from glycogenolysis, and it acts as a glucose pool prior to the onset of hepatic gluconeogenesis [154].

In comparison, the increase in hepatic PEPCK mRNA is very weak and not significant immediately, but it may also peak during recovery or after prolonged exercise, when glycogen stores are depleted and hepatic gluconeogenesis is the only source of glucose. Irisin Reduces Hepatic Glycolysis via PI3K-AKT-Fox01 Downregulation of PCK1 and G6PC in Primary Hepatocyte Cultures of Mice in IR [155]. Researchers down-regulated FNDC5/Irisin protein levels in liver and serum by long-term high-fat diet and inhibited the activation of Akt-Fox01 pathway phosphorylation, leading to an abnormal increase in hepatic gluconeogenesis, suggesting that FNDC5/Irisin may be a potent target for exercise to inhibit insulin resistance-associated hepatic gluconeogenesis and ameliorate chronic metabolic disorders such as depression [118,155,156]. It has been found that Irisin, produced by exercise-induced skeletal muscle contraction, regulates brain-derived neurotrophic factor (BDNF) levels in the brain through the peripheral pathway, which in turn regulates mood, cognition and other functions [157,158]. Liver gluconeogenesis can maintain glucose levels within a normal range without external glucose input, and glucose is the main energy supply for most cells. For example, the brain relies mainly on the energy produced by glucose to maintain normal physiological functions [17,159]. Ling-Hu used stable isotope tracing metabolomics and found that the isotopic abundance of the final product of glycolysis (pyruvic acid) in the hippocampus of CUMS depressed rats decreased, while the key enzymes of gluconeogenesis (PC and PEPCK) had high activity in CUMS depressed rats, which was one of the reasons for this result. This indicates that the decrease in isotopic abundance of pyruvic acid is not caused by glycolysis but may be the result of accelerated gluconeogenesis metabolism [52]. Wang Xianxian found in experimental research that Xiaoyao San can exert antidepressant effects by reversing the abnormal expression of PCK1 and G6pase, allowing pyruvate to re-enter the TCA cycle and restore normal metabolism of the TCA cycle and liver gluconeogenesis [48].

In summary, hepatic gluconeogenesis key rate-limiting enzymes, PEPCK, G6Pase, FBP and PC, regulate the hepatic gluconeogenesis process through hormone-regulated transcription factors and coactivators, including CREB, LKB1, Fox01, HNFα and PGC-1α. A study was conducted from the perspective of the muscle-brain axis and found that depression was associated with abnormalities in skeletal muscle mitochondrial unfolded protein (UPRmt) and lactate metabolism (Figure 1), which may affect brain function through monocarboxylic acid transporter protein 1 [36,160]. From a gut–brain axis perspective, depression has been found to be strongly associated with disturbances in gut flora metabolites (short-chain fatty acids) and pentraxin (5-HT) signaling [161]. From the perspective of the mind-brain axis, inflammatory factors (interleukin-6, interleukin-1β) have been found to affect synaptic plasticity through the TLR4/NF-κB pathway, which is associated with the development of depression [36,162,163,164]. And this article focuses on the perspective of the liver–brain axis, exercise-induced lactate was found to maintain glucose homeostasis through a series of regulatory transcription factors that modulate the core rate-limiting enzymes of hepatic gluconeogenesis (PEPCK, G6Pase).

## 6. Summary and Outlook

In summary, exercise regulates liver gluconeogenesis, metabolic substrates, and key metabolic enzymes, thereby affecting glucose production, providing a more stable energy supply to the brain, improving cognitive and emotional functions, and enhancing liver insulin sensitivity, maintaining blood glucose homeostasis, and reducing emotional fluctuations caused by insufficient blood glucose. In terms of physiological mechanisms, hepatic gluconeogenesis may be one of the potential targets for exercise intervention in depression, which provides a theoretical basis for clinical exercise therapy to improve depressive symptoms. Furthermore, this review adopts the framework of bidirectional communication within the liver–brain axis and draws on the systematic perspective of existing reviews on this axis. However, unlike their panoramic descriptions of multiple pathways, this review limits its analysis to the specific sequential link between exercise—liver gluconeogenesis—brain energy metabolism—emotional regulation. Although the literature has identified multiple signaling pathways through which exercise regulates hepatic gluconeogenesis, the direct causal link between exercise-induced hepatic gluconeogenesis and emotional improvement remains weak in the context of depression, and there is a lack of specific knockout animal models or intervention studies to validate the necessity of this axis. Future studies should focus on personalized exercise interventions for metabolic regulation to optimize depression treatment; integrate stable isotope tracing and multi-omics techniques to reveal the spatial-temporal dynamic mechanisms of liver-derived metabolites in central mood regulation, and promote integrated research in liver metabolism-depression and other psychiatric disorders.

It should be noted that this review has the following limitations: As a narrative review, although the search covered multiple Chinese and English databases, the scope of the search may still be biased. It may have omitted some classic studies and did not cover relevant findings published in other languages. Additionally, the inclusion of the literature primarily focused on peer-reviewed journal articles, without systematically incorporating books and the gray literature, may have resulted in incomplete coverage of literature types. Although the retrospective literature searches supplemented these omissions, comprehensive inclusion of all critical studies cannot be fully guaranteed. Overcoming these limitations in future research may position hepatic gluconeogenesis as a pivotal bridge linking metabolic regulation and psychological interventions, potentially opening new pathways for the application of exercise therapy in depression treatment.

## Data Availability

All graphic and textual materials generated or analyzed in this study are included in this article.

## Abbreviations

The following abbreviations are used in this manuscript:

ACOT12	Acyl-CoA Thioesterase 12
CUMS	Chronic Unpredictable Mild Stress
CSDS	Chronic social defeat stress
TCA	Tricarboxylic Acid Cycle
CREB	Cyclic adenosine monophosphate response element binding protein
G6Pase	Glucose-6-phosphatase
GABA	Gamma-Aminobutyric Acid
HNF4α	Hepatocyte Nuclear Factor 4 Alpha
IL-6	Interleukin-6
IL-Iβ	Interlenkin-Iβ
LDH	Lactate dehydrogenase
LKB1	Liver kinase B1
PC	Pyruvic carboxylase
PK	Pyruvate kinase
PCK1	Phosphoenolpyruvate carboxykinase
PEPCK	Phosphoenolpyruvate carboxykinase
PGC-1α	Peroxisome proliferator-activated receptor-gamma coactivator-1 alpha
SCFAs	Short-chain fatty acids
5-HT	5-Hydroxytryptamine
MCT1	Monocarboxylate transporter 1
